# Health and demographic surveillance systems: a step towards full civil registration and vital statistics system in sub-Sahara Africa?

**DOI:** 10.1186/1471-2458-12-741

**Published:** 2012-09-05

**Authors:** Yazoume Ye, Marilyn Wamukoya, Alex Ezeh, Jacques B O Emina, Osman Sankoh

**Affiliations:** 1 ICF International, 11785 Beltsville Drive, Suite 300, Calverton, MD, 20705, USA; 2 African Population and Health Research Center, Nairobi, Kenya; 3 Department of Population and Development Studies, University of Kinshasa, Kinshasa, Democratic Republic of Congo; 4 CEPS/INSTEAD, Esch-sur-Alzette, Luxembourg, Luxembourg; 5 INDEPTH Network, Accra, Ghana; 6 School of Public Health, University of the Witwatersrand, Johannesburg, South Africa; 7 Institute of Public Health, University of Heidelberg Medical School, Heidelberg, Germany

## Abstract

**Background:**

In the developed world, information on vital events is routinely collected nationally to inform population and health policies. However, in many low-and middle-income countries, especially those in sub-Saharan Africa (SSA), there is a lack of effective and comprehensive national civil registration and vital statistics system. In the past decades, the number of Health and Demographic Surveillance Systems (HDSSs) has increased throughout SSA. An HDSS monitors births, deaths, causes of death, migration, and other health and socio-economic indicators within a defined population over time. Currently, the International Network for the Continuous Demographic Evaluation of Populations and Their Health (INDEPTH) brings together 38 member research centers which run 44 HDSS sites from 20 countries in Africa, Asia and Oceana. Thirty two of these HDSS sites are in SSA.

**Discussion:**

This paper argues that, in the absence of an adequate national CRVS, HDSSs should be more effectively utilised to generate relevant public health data, and also to create local capacity for longitudinal data collection and management systems in SSA. If HDSSs get strategically located to cover different geographical regions in a country, data from these sites could be used to provide a more complete national picture of the health of the population. They provide useful data that can be extrapolated for national estimates if their regional coverage is well planned. HDSSs are however resource-intensive. Efforts are being put towards getting them linked to local or national policy contexts and to reduce their dependence on external funding. Increasing their number in SSA to cover a critical proportion of the population, especially urban populations, must be carefully planned. Strategic planning is needed at national levels to geographically locate HDSS sites and to support these through national funding mechanisms.

**Summary:**

The paper does not suggest that HDSSs should be seen as a replacement for civil registration systems. Rather, they should serve as a short- to medium-term measure to provide data for health and population planning at regional levels with possible extrapolation to national levels. HDSSs can also provide useful lessons for countries that intend to set up nationally representative sample vital registration systems in the long term.

## Background

### Civil registration and vital statistics system (CRVS)

#### Informing policies with strong evidence

The main purpose of a civil registration and vital statistics system (CRVS) is to record the occurrence of key (or vital) events in the lives of the people in a given geographic area. In 2003, the United Nations defined these as comprising a live birth, death, fetal death, marriage, divorce, adoption, legitimisation of birth, recognition of parenthood, annulment of marriage, or legal separation [1]. The greatest advantage of a CRVS is that it is continuously collecting data and therefore, it is possible to study the changes in a population’s vital events over a period of time. The earliest CRVS was in the form of church or parish registries in Europe (as early as the 14th century) and Japan, which monitored births, deaths and marriages in separate registers. These evolved into civil registries or CRVS in the 19th and 20th centuries [1].

The best CRVS is mandatory, permanent and continuous. In addition, it should be complete, in that it registers all vital events and it must provide high quality data in a timely manner. Completeness can be measured by comparing the percentage of recorded vital events with an estimate that has been established independently for that population. Some of the best CRVSs can be found in developed countries; one example of such a CRVS is the national registry of England and Wales, established by a Parliamentary Act in 1837. In Australia, each state and each territory maintains its own registry. In the United States, the vital registry is the purview of each state and in some cases, of a city. Several countries in Central and South America have a complete CRVS. In Asia, Sri Lanka stands as the one of the few nations with comprehensive CRVS (more than 90% of the events covered) that is compulsory [1]. While some CRVSs are not complete, they have been established by law to maintain specific aspects of a registry. An example is the Maldives, where in July 1993, a law was enacted requiring all births and deaths to be certified and registered by the Ministry of Health [1-4].

The information contained within the CRVS is a legal record that documents each vital event and defines a person’s entitlement to certain rights within a population. The statistics formed by aggregating these records reveal trends in the population. They can be used to evaluate the needs of a population so as to set up programmes that will meet these needs [1,5,6].

Vital statistics are used to monitor levels and trends in the fields of population, public health and administrative planning. For public health decision-makers and policy promulgators to be effective, they require access to timely and complete data on births, deaths, causes of deaths and illness in their jurisdictions and CRVS has proven to be a reliable source of these data. The data collected by CRVSs can also be used directly to monitor and evaluate the impact of any public health initiative that has been undertaken by governing bodies to improve the quality of health in their communities [1,5-9].

#### Vital registration in sub-Saharan Africa

CRVS is either non-existent or incomplete in most of sub-Saharan Africa (SSA). In many instances, although vital registration is conducted, there is inefficiency in the compilation of vital statistics, resulting in the paucity or poor quality of the data generated. In countries like South Africa and Zimbabwe, studies of death were carried out using civil registration data of deaths in selected cities in each country [10]. In most cases, the deaths registered are not medically certified and in fact, deaths due to HIV/AIDS are often attributed to other causes [10]. As such, statistics regarding adult mortality in SSA are calculated largely using prediction models and estimation procedures. It should be noted that South Africa is making strides to generate fairly reliable vital statistics from its CRVS and Statistics South Africa (StatsSA) plays a leadership role in promoting the development of CRVS in SSA. The World Health Organization (WHO) states that there are only five African countries in which there are vital registries that collect data on more than 25% of the population. This means that most people in SSA are born, live, and die without any record of any of these or other events in their lives [6,10-12]. According to the United Nations (UN), few countries in SSA use civil registration as the source of the vital events data reported [13,14]. According to UN Statistics, among the 14 countries which cite civil registration as the source of vital data, only four have more than 90% coverage of the events and these countries are mostly islands (Table 1). 

**Table 1:** Civil registration as a source for data on live births and deaths submitted to the United Nations between 1996 and 2010 by various sub-Sahara African Nations

**Country**	**Data on live births**			**Data on deaths**		
	**Source of data VRS?**	Level of completeness*	**Year of reference**	**Source of data VRS?**	Level of completeness*	**Year of reference**
Botswana	Yes	<90%	2006	Yes	<90%	2007
Cape Verde	Yes	>90%	2010	Yes	>90%	2010
Congo	Yes	<90%	2004	No	-	-
Djibouti	Yes	<90%	1996	No	-	-
Ghana	Yes	<90%	2008	No	-	-
Kenya	Yes	<90%	2009	Yes	<90%	2009
Lesotho	Yes	<90%	1996	No	-	-
Mali	Yes	<90%	2001	No	-	-
Mauritius	Yes	>90%	2010	Yes	>90%	2010
Reunion	Yes	>90%	2007	Yes	>90%	2007
Rwanda	Yes	<90%	2009	Yes	<90%	2009
Seychelles	Yes	>90%	2010	Yes	>90%	2010
South Africa	Yes	<90%	2009	Yes	<90%	2009
Zambia	Yes	<90%	2006	Yes	<90%	2006

* > 90% = More than 90% of the events covered; <90% = Less than 90% of the events covered.

Note:

· The UN used the latest data available from each country (data was from 1996 – 2010) to generate indicators on live births and deaths.

*·* T*hese statistics are based on country self-reported information.*

Source: http://unstats.un.org/unsd/demographic/products/vitstats/Sets/Series_A_2012.pdf.

The lack of an effective civil registration system (CRS) and CRVS presents immense challenges for evidence-based health and population policies. Political will, instability (war), lack of adequate resources, a culture of non-data-use, lack of an effective evidence based-decision making process are some of the issues that explain the lack of viable CRS and VRS in SSA [1,7,12,15].

#### Political will and commitment

In most countries in SSA, CRVS is not the first priority in the political agendas and ministries do not approach registration systems in/when seeking data that they can use in their decision-making processes. The International Institute for Vital Registration and Statistics (IIVRS) found that the biggest disincentive to establishing and running CRVSs in the late 1970s was a lack of commitment on the part of the political class [7]. The critique on political will is caused by an over-reliance on the Ministries of Health to contribute to data collection and analysis. These ministries usually focus on service provision rather than on making investments on population-based data collection and analysis. Consequently, there is need for a national dialogue involving key stakeholders who use vital statistics in their various businesses.

#### Instability and war

For the past decades, many countries in SSA have experienced civil wars. Countries like Angola, Cote d’Ivoire, Ethiopia, Liberia, Mozambique, Namibia, Rwanda, Sierra Leone, South Africa (Apartheid), and Uganda have been engaged in civil wars. Others like Chad, DRC, Somalia, and the Southern Sudan and Sudan continue to be ravaged by civil wars. Wars in SSA have destroyed the infrastructure and depleted human resources through disabilities, death, and displacement. These conditions make it virtually impossible to run any kind of effective registration system particularly in the face of massive internal isplacement of people fleeing the wars [15]. However, irrespective of the civil wars in some of the countries, efforts are ongoing in many others to generate vital statistics.

#### Lack of adequate financial resources

CRVSs are expensive to establish and maintain. For example, in 2000, Chile projected the annual cost of its CRVS to be USD 45 million of which 80% was generated by the system itself. This was done by attaching fees to services like issuing certificates, drivers’ licenses, and passports. Taxes raised by Chile’s CRVS provided the other 20%. Allocation of financial resources is another factor to consider. In Tanzania, in the 1990s, USD 30 million was used to gather health data using various systems but very little of it (USD 700,000) was spent in maintaining its CRVS; no population estimates were generated from the data it collected [7]. While Chile has shown that it is possible to finance a CRVS, the Tanzanian example shows competing interests might derail the process. Financing and allocating resources for a CRVS hence need to be carefully thought out and Ministries of Finance and Planning should be effectively be brought on board at the outset.

#### Culture of non-data use

There has been little evidence-based decision making in most of SSA for many reasons, one of which is that the available data may not be aligned with the political views or interests of potential users. If there was high demand for data for decision-making, there would be greater incentive to put in place a system such as CRVS to generate quality and reliable information [7]. This is however not the entire story. The lack of the requisite local capacity to collect, curate, analyse and publish data contributes significantly to desire not to collect and make no use of the data. It is therefore extremely important to introduce strategies that would cater not only for data collection but also for their preparation, analysis and use.

Given the above thorny issues that must be dealt with, it is therefore clear that it will be some decades before full registration systems are established in SSA [1,12]. The need for a viable alternative that provides a reliable source of vital data is critical and the emerging health and demographic surveillance systems (HDSSs) in LMICs could go a long way in addressing the chronic data gaps in SSA.

### Health and demographic surveillance systems (HDSSs)

#### Definitions and concepts

**Figure 1:**
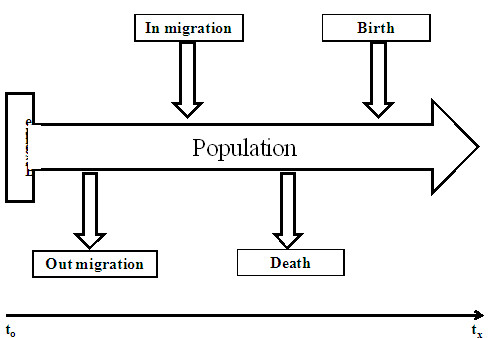
The structure of a health and demographic surveillance system.

An HDSS consists of monitoring demographic and health characteristics of a population living in a well-defined geographic area [16-18]. The process starts with a baseline census followed by regular update of key demographic events (birth, death and migration) and heath events. Figure 1 presents this HDSS concept. A typical HDSS may also include registration of marriages, divorces, changes in status and household relationships and fertility estimates. Such additional information is critical for a better understanding of the health and demographic dynamics of the population under observation. With such a dynamic platform that can monitor new health threats, can track population changes through fertility and migration rates, and can measure the effect of interventions on communities, the HDSSs can be used to generate evidence to guide health policy making. 

HDSSs provide complementary and in some cases intermediate data to the other well-known methods, such as national censuses, and demographic and health surveys (DHSs). The main difference is that whereas HDSSs collect data prospectively and longitudinally on a district, for instance, censuses and DHSs collect data on a cross-sectional basis at generally long intervals (10 years for the census and 3–5 years for DHS) on either the whole population or on large nationally representative samples. The complementarity of HDSS data lies in the fact that the platform can be used to evaluate the population impact of health interventions at a community level and produce health and population indicators for the periods between two censuses or DHSs. Furthermore, the HDSS population can be used as a sampling frame for other studies and surveys. For example, morbidity, livelihood, and household panel surveys and cohort studies can use HDSSs as sampling frames allowing the surveys to be carried out within the rounds of the HDSS.

Data on causes of death provide critical statistics for policy makers at every level of governance –from local to national – to prioritise and address the health needs of their people. However, this information is not readily available in most low-income countries. In high-income countries, information on cause specific mortality data is usually made available through CRVSs linked to the health care system [3]. Unfortunately, in low-income countries, most deaths occur outside the health care system, making it difficult for a CRVS to obtain medically certified cause of death [4]. To address this challenge, the Verbal Autopsy (VA) [19] method was included in HDSSs to provide valuable information on patterns of causes of death. Despite some concerns (low sensitivity and specificity for some diseases) regarding this approach, there is general agreement that VAs are the best alternative method available for low-income countries to understand cause of death patterns in the absence of national vital registration systems. A very good development in VA use is a shift from using physician coding to arrive at cause of death to an automated procedure like Inter VA [20-22] which is cheaper, faster and more consistent. The INDEPTH Network is working together with WHO and other organisations to make faster progress in this area.

#### HDSS - brief history and current situation

The establishment of HDSSs started in the 1940s in SSA, with the Pholela Health Centre in South Africa being the first site created in 1940. This is a Community Oriented Primary Health Care programme established by the South African Ministry of Health to prevent and treat diseases prevalent in rural Natal (such as tuberculosis, syphilis, smallpox, typhoid, and measles), and to inform health policy [23-25]. Niakhar in rural Senegal is the second site and was created in 1962 under the Niakhar Project [26]. Unlike Pholela, it was independent from the national Ministry of Health. Among other contributions, Niakhar has provided insight into the effectiveness of several public health interventions [27-32].

Pholela and Niakhar have paved the way for numerous health research stations across the continent. Among these, the most acclaimed worldwide are Danfa in Ghana [33], Kasongo in the Democratic Republic of Congo [34], Kilombero in Tanzania [35], Agincourt in South Africa [36], Nouna in Burkina Faso [37], and Navrongo in Ghana [38]. With the increasing need to tackle new pandemics such as HIV/AIDS, to address the African urban health crisis, while devising strategies for health planning at the district level, a new generation of HDSS-based field stations have recently emerged, including Hlabisa (now Africa Centre) in South Africa, Nairobi in Kenya, and Rufiji in Tanzania.

More HDSS sites have been established within the past 40 years in areas that do not have a functional VRS. They may have been instituted differently but they all track population and demographic changes within their research sites or surveillance areas. Today, there is an international network of independent research centres that run HDSSs mainly in SSA, Asia and Oceania. The INDEPTH Network (http://www.indepth-network.org) was established in 1998 and currently has a membership of 36 member centres which run 44 HDSSs with 32 of the HDSS in SSA [6,12,17,39]. In a recent editorial (These HDSS sites are presented as a series of cohort profiles which follows a similar structure to forthcoming profiles [40].

The INDEPTH Network provides an environment in which researchers from the HDSS sites can jointly collect data and compare findings mostly through working groups formed around specific research agendas. The network offers a unique platform covering a total population of over 2.4 million individuals in Sub –Sahara Africa. Details on population coverage per country and HDSS site are presented in Table 2.

**Table 2:** Distribution of HDSS sites registered as member as per 2011 and the population they cover in sub-Saharan Africa

**Country**	**Number of HDSS in the country**	**Urban/Rural site**	**HDSS population**	**HDSS coverage of country population (%)**
Burkina Faso	5	Rural, Semi-urban	432,000	2.8
Cote d'Ivoire	1	Rural	37,000	0.2
Ethiopia	1	Rural	60,000	0.1
Ghana	3	Rural	396,000	1.7
Gambia	2	Rural	56,000	3.3
Guinea-Bissau	1	Rural, Semi-urban	105,000	6.2
Kenya	4	Rural, Urban	592,000	1.6
Malawi	1	Rural	33,000	0.2
Mozambique	1	Rural/Peri-urban	86,000	0.4
Senegal	3	Rural	69,200	0.5
South Africa	3	Rural, Peri-urban	184,000	0.4
Tanzania	3	Rural	277,000	0.7
Uganda	2	Rural, Peri-urban	124,000	0.4
**Total**	**30**		**2,451,200**	**0.7**

**Source: INDEPTH Network web page:**http://www.indepth-network.org.

#### Some key benefits of HDSSs in providing population-based data

**Figure 2:**
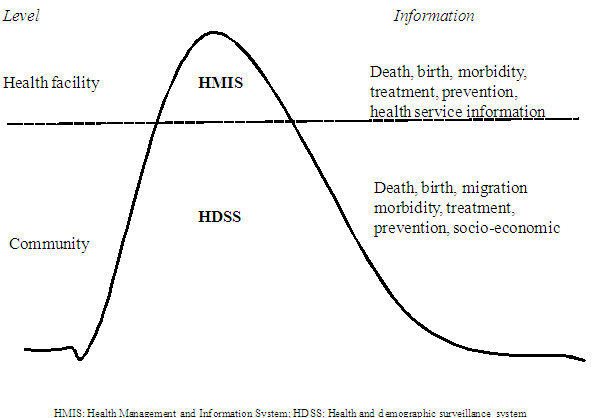
Complementary roles played by HDSS and HMIS.

Many SSA countries had centralised health management and information systems (HMISs) which focused on morbidity and mortality reporting. Data flew from individual health units to the district and national levels. In the early 1990s, the Ministries of Health tried to implement HMISs which focus on using information at the point of collection [41]. However, since HMISs provide data that are based on information from users of health services, they show part of the picture because of the low coverage and use of health care in SSA (Figure 2). 

To complement HMIS, data generated from HDSSs can be used to provide population-based data that can inform health systems decisions and performance. This is because, as mentioned before, HDSSs monitor vital events and other socio-economic data at the community level.

They provide accurate information that can be used to promulgate evidence-based health policies and then to monitor the impact of these policies on populations [6]. For instance, a study that compared mortality estimates of deaths by passive government surveillance and active HDSS in Western Kenya found that although there was under-reporting of peri-natal deaths, the distribution of child deaths was similar across the two systems for children aged 1 – 59 months. The authors also found a significant difference in cause of death between the two systems with the civil registry under-reporting malaria and pneumonia and over-reporting measles [42].

Another example of the benefits of HDSS in determining health service needs is the Tanzania Essential Health Interventions Project (TEHIP) model of Rufiji and Morogoro in Tanzania. In 1996, both the national statistics and the district statistics for Morogoro indicated that malaria was the number one reason for visits to health facilities. This came as no surprise to national and district level health service planners. In that year’s district health plan, when health programmes were being implemented, malaria was not singled out as a priority. A sentinel HDSS in the rural district found that 30% of total mortality burden was due to malaria, and 45% of mortality in children younger than five was due to malaria. They also found that 46% of deaths occurred without prior visits to a health facility. Therefore, the district had underestimated both the number of people who visited health facilities for malaria and the disease burden of malaria. Because of the HDSS data, the district reallocated its resources in its health plans so that there was a five-fold increase in resources being allocated to malaria control and a 20-fold increase in resources to malaria control in children younger than five.

The HDSS was also instrumental in evaluating the impact of two programmes that were being implemented by the district, i.e. the Integrated Management of Childhood Illness (IMCI), a strategy that was devised by WHO, and the use of insecticide-treated bed nets (ITNs) [43,44].

#### Some key challenges of HDSSs

Centers running HDSSs are funded in several ways. Some are funded by private institutions, foundations and international public funds. There is an increasing number of them funded by in-country institutions such as universities. However, there is need to connect research at some of these HDSSs with national interests and priorities in order to increase interest from governments in supporting the HDSS sites and in using research findings from the systems. The focus on publishing results more in international peer-reviewed journals should be juxtaposed with a strategy to engage in national policy dialogue. The INDEPTH Network is trying to address this challenge by working with the centers to synthesise research results into policy briefs and specially written reports which address national priorities.

HDSSs are geographically localised, mostly in rural areas, and the population covered is in most cases not representative of the entire geographic area or is often small, to minimise operational costs. It has therefore been argued that one cannot use measures obtained from HDSS data to make inferences about other regions or at the national level. However, a historical look at the designing and validating of epidemiological surveillance in uncounted populations in Sweden concluded that “Unsubstantiated claims that local sub-national population data are “unrepresentative” or “only local” should not therefore predominate over likely representativity” [45]. Further, a comparison of HDSS and DHS approaches to mortality surveillance in Ethiopia revealed comparable overall mortality patterns. However, HDSS data were more sensitive to local variations and less subject to recall bias [46].

It has also been suggested that a population under such regular and intensive surveillance such as the one under an HDSS may have better health indicators compared to a population from another setting because the repeated measurement is seen as a sort of passive intervention. In addition, populations from HDSS areas are often exposed to studies that may provide interventions (e.g. clinical trials, testing an intervention, among others). Thus, indicators from HDSS sites should be extrapolated to other settings with caution. However, there is need for a detailed investigation to assess how different the population in an HDSS is, from the population in a non-HDSS setting [46]. It is also important to explore possible statistical techniques using data from different sources to correct for potential differences between HDSS populations and other populations.

As HDSSs continue to be run over a long period, a number of operational challenges and constraints are to be expected. For instance, respondent fatigue has been a case in point especially for HDSSs that update the population data at least three times a year. But HDSS managers have found solutions to this challenge since many of the systems have existed over decades and will continue to do so. Investigators from the community are used and the individuals are not always the same study subjects in nested projects within the HDSS. In addition, some centers provide health intervention to the community. For example, the African Population and Health Research Centre (APHRC) running the Nairobi HDSS provide free health camps to the population under surveillance twice a year.

Another challenge is the cost of operating the core HDSS. For example, a survey conducted by INDEPTH in 2009 put an average of USD 250,000 per year for an HDSS of 60,000 people with three update rounds per year in rural settings. This gives a per capita cost of USD 4.17. When this cost is compared with the value of the data generated and the platform created for a host of other studies, an HDSS can be considered cost effective. Funders who are reluctant to fund the surveillance infrastructure must be educated better on the utility of the platform. A few funders have started to agree to provide some support for running the core HDSS in INDEPTH projects they fund. Governments should also be made to appreciate the value of such systems and to contribute to the running cost of HDSSs in their countries. It is worth mentioning that Tanzania, Ghana and Burkina Faso are examples of countries where the Ministries of Health contribute in small but significant ways to the running of HDSSs in their countries. The INDEPTH Network will need to do more advocacies and the HDSS centre leaders will have to engage more in projects that will make their systems more relevant to the national agendas through direct engagement with the Ministry of Health.

HDSSs have faced challenges in providing timely data such as cause of death so that they can be used in annual sector reviews. This is largely due to the complex relational and longitudinal data generated by the HDSSs which require advanced demographic/statistical analyses, and less to do with the uninformed calls to share data irresponsibly. More capacity needs to be developed and strengthened in data management and analysis. Thanks to a few funders who are convinced about the role of INDEPTH in this area, the Network is focusing its energy to increase data management and analysis capacities in the South. INDEPTH contributed data to the Global Burden of Diseases Estimates by WHO and several datasets are now made freely available on the Network’s website: http://www.indepth-network.org and an online repository (iSHARE) has been created to share HDSS data: http://www.indepth-ishare.org.

## Discussion

### How can HDSSs help to build national vital registration systems

HDSSs can help to build CRVSs by providing experience in vital registration operational techniques. As both systems are longitudinal in nature, HDSSs provide an appropriate platform for the training of personnel in order to enable them generate and manage longitudinal databases.

HDSSs can help in building and retaining capacity on technical skills pertaining to data collection, management, quality control, archiving and retrieval, analyses and dissemination when other capacity building programmes seem to face challenges. This will enhance the use of the data in active research, and networking of national experts working on similar issues.

### Promoting data use

The culture of increased data use and evidence-based decision making can be strengthened by using data generated by HDSSs to produce, present, and/or publish indicators to governing bodies and peer-reviewed journals so that these indicators may be used to arrive at decisions that improve public health. Two notable examples of the use of HDSS-generated evidence can be found in Ghana and Malawi. In Ghana, evidence of the impact of the Community-based Health Planning and Services initiative (CHPS) at the Navrongo HDSS site has resulted in a scale up of the initiative. In 1994, a pilot study was conducted at this site to develop a model of a community-based service delivery system and test its efficacy, comparing it to static-based services. The major facets of this initiative that were considered were the cost-effectiveness of care, the receptiveness of the community, and the mobilisation of the community members to take an active role in their own health management and care. The Ministry of Health, upon learning of the efficiency of CHPS, adapted this initiative in at least one district in each region of Ghana by scaling it up in order to change the manner in which health care services were delivered, especially in rural areas. Over the years, CHPS was scaled up to cover more geographical regions in Ghana [47,48].

The Karonga HDSS in Malawi showed that all-cause adult mortality fell by 32% at the population level in the three years following the initiation of antiretroviral therapy roll-out in the district in 2005. This was attributable to a decline in AIDS related deaths and reversed the trend of adult mortality associated with the HIV epidemic [49,50]. Integration of the HDSS geographic information system data further demonstrated that the decentralisation of services to peripheral health facilities improved the benefits for remote communities [49]. This provides strong support for the public health approach to antiretroviral therapy (ART) delivery advocated by WHO [51], and the data were used to support renewal of Global Fund awards to Malawi, further funding the ART roll-out programme.

### Creating the capacity for data processing

The management and analysis of cross-sectional data are standard in most academic institutions whereas complex longitudinal data generated by HDSSs always pose challenges. Special expertise in relational longitudinal data analysis is required to handle HDSS data. Hence HDSSs can be used to create the capacity for the generation, management and analyses of longitudinal data since the centers often employ demographers, epidemiologists, sociologists, clinicians, and economists, these staff can train other professionals on the generation and management of such data [52].

### Creating demand and value for such a system

As more and more governing civil entities use HDSS data to make decisions that affect their communities, the usefulness of HDSSs will become more apparent to the government, which in turn will create the demand for this kind of data. As a result, governments will more likely become increasingly involved in the day-to-day operations of HDSS and will encourage the establishment, maintenance and funding of such sites at strategic locations to provide more of the same data from several areas in their regions. Examples are Dodowa, Kintampo and Navrongo HDSSs in Ghana which owned by the Ghana Health Service [53], the Manhica HDSS in Mozambique, which is was the first research center the Ministry of Health established peripherally [54], the Kisumu and Kilifi HDSS sites in Kenya which are both co-managed by the Kenya Medical Research Institute (KEMRI) [55,56], the Nouna HDSS in Burkina Faso which is partly funded by the Ministry of Health [57] and the Bandim HDSS in Guinea-Bissau, which collaborates with the Ministry of Health [58].

### Setting the stage for sample registration system?

HDSSs can be used to build a national CRVS through the creation of a sample vital registration (SVR), which is essentially a collection of many HDSSs with exclusive focus on the vital events. HDSSs provide the capacity for generating data and could function as a starting point [59].

An SVR is longitudinal like an HDSS that registers events and counts the population among which the events occur. The difference is that an SVR covers many larger areas that are sampled sequentially from an entire geo-political region, like a province, or a country. The SVR monitors births, deaths and migrations in a sample of individuals, households and/or residential units in a specific area and is able to provide accurate estimates for the population that is at risk. In China, mortality statistics that are representative of the population are obtained from SVR. China and India have implemented SVR nationally and two Asian and four African countries are planning to start such a system [59].

In addition, a full-fledged HDSS can be operated within or around the SVR in order to collect more detailed information such as migration patterns, health and disease burdens, and several other socio-economic factors. Such an arrangement would allow for the HDSS and the SVR to validate and complement each other [16].

An SVR/HDSS combination may be inexpensive to maintain than maintaining both side by side and may bring the benefits of nationally representative vital events statistics along with the detailed data for health intervention evaluation. However a detailed investigation of the merits and demerits of this combination needs to be carried out find out the most cost effective and efficient approach.

## Summary

### When will CRVSs become widespread in SSA?

There is a lack of CRVS in Africa, and in particular SSA, yet this region needs surveillance more than any other to monitor and understand health and population issues in order to inform policy. The factors that have contributed to this situation include lack of commitment and will from the political class, instabilities and wars which have the effects of displacing individuals and governing bodies, financial constraints, and non-appreciation of the importance of the CRVS-generated data in informing policies.

It is widely accepted that a CRVS is important for evidence-based decision making and its lack has created a data gap on population, health and other critical sectors. Given the challenges it is likely to take decades before SSA countries have established CRVS that is similar to the compulsory CRVS in Western nations. There is therefore a need to consider HDSSs as stepping stones to CRVSs in LMICs.

In order to answer the question we have asked, when will CRVS be widespread in SSA, there should be a discussion by the national statistics offices on the concern in relation to the cost of SVRs, who should fund SVRs and whether or not SVRs compete with the development of CRVS. A fuller discussion is subject of another paper.

### How do HDSSs increase their role in national statistics?

The purpose of an HDSS is to generate longitudinal information on the health and population of a geographically defined population. Several member centers of INDEPTH have established their HDSS sites to generate this information and guide policy making. The data collected from these sites have been instrumental in the launching of various national health programmes across SSA. It should be noted that a larger number of HDSSs in a country is better than having just one and collaboration among these HDSSs will strengthen the role they collectively play in contributing to national statistics. This is happening now in Ghana, Kenya, South Africa, Burkina Faso and Bangladesh where there are at least three HDSSs in each country.

A recent discussion with members of the INDEPTH Scientific Advisory Committee concluded that for HDSS information to be used for national statistical and health systems, some of the following barriers and constraints need to be overcome:

• HDSSs currently influence national policies largely indirectly through partners rather than directly through national authorities. If HDSS results are to be used directly in national statistics and information for policy, they need to be viewed more clearly as national resources that generate information of national policy relevance.

• Planning for additional HDSS sites with the aim of achieving a better geographic balance could help enhance the perception of national statistics offices as being nationally relevant.

• To counteract the perception on the part of national authorities that the vital statistics and other indicators from HDSS sites are not representative of the population as a whole, HDSSs should support the tracking of key indicators in other parts of the country for comparative purposes and use the findings to correct for potential bias in HDSS statistics and thus render them more relevant for the country as a whole.

• Working more closely and directly with national authorities to identify priority activities could ensure that HDSSs address national research priorities and enhance national ownership and support.

• For HDSS to be perceived as integral to national statistical systems and policy making, they should be positioned not as ends in themselves or instruments for generating research findings for external agencies, but as temporary mechanisms for generating national data relevant to national health, population and development priorities.

• HDSS could serve as a resource to country statistical and policy systems by partnering HDSS staff with staff from national programmes. HDSS have the advantage of highly skilled staff experienced in the generation, manipulation and dissemination of demographic and epidemiological data. These staff could mentor those working in national statistical and health information offices and provide direct assistance in data analysis and data quality assurance.

• A strong potential advantage of HDSSs is their promotion of a culture of data use and evidence-based decision making. It is important to build upon this to achieve improvements in data dissemination and use at national level also. People working in HDSSs often have skills in presentation, dissemination and publication in peer-reviewed journals that can be shared with those working in national health information and statistical systems where data dissemination capacities tend to be limited.

On the whole, for HDSSs to continue playing a key role and ultimately help in building CRVSs in the South, there is need for greater commitment and involvement by the government in countries where these systems currently operate.

## Competing interests

Osman Sankoh is the Executive Director of the INDEPTH Network. All other authors declare no competing interest.

## Authors’ contributions

YY conceptualised and wrote the first draft of the manuscript. MW, AE, JE and OS participated substantially in the write up and revision of the manuscript. All authors read and approved the final manuscript.

## Pre-publication history

The pre-publication history for this paper can be accessed here:

http://www.biomedcentral.com/1471-2458/12/741/prepub

## References

[B1] Lucas D Meyer P Registration, Administrative, and Qualitative Data Beginning Australian Population Studies 2003 2 National Centre for Development Studies, Research School of Pacific Studies, Australian National University, Australia 1 8

[B2] Weed JA Vital statistics in the United States: preparing for the next century Popul Index 1995 61 4 527 539 10.2307/3645558 12347152

[B3] Status of Mortality Statistics in the Maldives http://www.searo.who.int/LinkFiles/2007_MortalityStatus-maldives.pdf

[B4] Mahapatra P Shibuya K Lopez AD Coullare F Notzon FC Rao C Szreter S Civil registration systems and vital statistics: successes and missed opportunities Lancet 2007 370 9599 1653 1663 10.1016/S0140-6736(07)61308-7 18029006

[B5] United Nations: Handbook on Civil Registration and Vital Statistics Systems Policies and Protocols for the Release and Archiving of Individual Records 2003 United Nations

[B6] Chandramohan D Shibuya K Setel P Cairncross S Lopez AD Murray CJL Zaba B Snow RW Binka F Should Data from Demographic Surveillance Systems Be Made More Widely Available to Researchers? PLoS Med 2008 5 2 e57 10.1371/journal.pmed.0050057 18303944 PMC2253613

[B7] AbouZahr C Cleland J Coullare F Macfarlane SB Notzon FC Setel P Szreter S Anderson RN Bawah AA Betran AP The way forward Lancet 2007 370 9601 1791 1799 10.1016/S0140-6736(07)61310-5 18029003

[B8] Mathers CD Fat DM Inoue M Rao C Lopez AD Counting the Dead and what they Died from: an Assessment of the Global Status of Cause of Death Data Bull World Health Organ 2005 83 3 171 177 15798840 PMC2624200

[B9] Lopez AD Counting the dead in China. Measuring tobacco's impact in the developing world BMJ 1998 317 7170 1399 1400 10.1136/bmj.317.7170.1399 9822388 PMC1114289

[B10] Timaeus IM Jasseh M Adult mortality in sub-Saharan Africa: evidence from Demographic and Health Surveys Demography 2004 41 4 757 772 10.1353/dem.2004.0037 15622953

[B11] Coale AJ Kirk D Hauser PM Grabill WH The Determination of Vital Rates in the Absence of Registration Data Milbank Mem Fund Q 1971 49 4 175 191 10.2307/3349470

[B12] Kahn K Tollman SM Collinson MA Clark SJ Twine R Clark BD Shabangu M Gomez-Olive FX Mokoena O Garenne ML Research into health, population and social transitions in Rural South Africa: data and methods of the agincourt health and demographic surveillance system Scand J Publ Health 2007 35 Suppl 69 8 20 10.1080/14034950701505031 PMC2826136 17676498

[B13] United Nations Population and Vital Statistics Report Demographic Yearbook Series A 2012 1 United Nations, New York 1 23

[B14] World Population Data Sheet 2008 http://www.prb.org/pdf08/08WPDS_Eng.pdf

[B15] The World Bank Group Transition from War to Peace in Sub-Saharan Africa Findings Africa Region Volume 81 1997 1 3

[B16] Clark SJ Evaluating the Performance of Demographic Surveillance Systems: Adult Mortality Adult Mortality in the Developing World: Methods and Measures: July 8th – 11th 2004 2004 Marconi Conference Center, California, USA

[B17] INDEPTH Network Sankoh OA DSS Concepts and Methods: Core Concepts of DSS Population and Health in Developing Countries. Population, Health and Survival at INDEPTH Sites 2002 IDRC, Canada 1 10

[B18] Sankoh O Binka F Becher H, Kouyate B INDEPTH Network: generating empirical population and health data in resource-constrained countries in the developing world Health research in developing countries: a collaboration between Burkina Faso and Germany 2005 Springer, Berlin 21 32

[B19] Setel PW Sankoh O Rao C Velkoff VA Mathers C Gonghuan Y Hemed Y Jha P Lopez AD Sample registration of vital events with verbal autopsy: a renewed commitment to measuring and monitoring vital statistics Bull World Health Organ 2005 83 8 611 617 16184280 PMC2626308

[B20] Fantahun M Fottrell E Berhane Y Wall S Högberg U Byass P Assessing a new approach to verbal autopsy interpretation in a rural Ethiopian community: the InterVA model Bull World Health Organ 2006 84 204 210 10.2471/BLT.05.028712 16583079 PMC2627286

[B21] Oti SO Kyobutungi C Verbal autopsy interpretation: a comparative analysis of the InterVA model versus physician review in determining causes of death in the Nairobi DSS Popul Health Metr 2010 8 21 10.1186/1478-7954-8-21 20587026 PMC2902422

[B22] Tensou B Araya T Telake DS Byass P Berhane Y Kebebew T Evaluating the InterVA model for determining AIDS mortality from verbal autopsies in the adult population of Addis Ababa Trop Med Int Health 2010 15 5 547 553 20214760 10.1111/j.1365-3156.2010.02484.x PMC3901008

[B23] Tollman S Kark S Kark E Das Gupta M, Aaby P, Garenne M, Pison G The Pholela Health Centre: understanding health and disease in South Africa through community-oriented primary care (COPC) Prospective Community Studies in Developing Countries 1997 Oxford, Clarendon Press 213 232

[B24] Chesler J Kark SL Survival in infancy; a comparative study of stillbirths and infant mortality in certain Zulu and Hindu communities in Natal S Afr J Lab Clin Med 1956 2 2 134 159 13360404

[B25] Kark S The Practice of Community-Oriented Primary Health Care: Appleton-Century-Crofts 1984 World Health Forum, New York 91 95 5

[B26] Garenne M Cantrelle P Das Gupta M, Aaby P, Garenne M, Pison G Three decades of research on population and health: the ORSTOM experience in rural Senegal, 1962–1991 Prospective Community Studies in Developing Countries 1997 Clarendon, Oxford 233 252

[B27] Rey M Mar Diop I Baylet R Cantrelle P Ancelle J Réactions cliniques au vaccin rougeoleux vivant atténué (Edmonston B) en milieu coutumier Sénégalais Bull Soc Méd Afrique Noire Langue Française 1964 9 255 271 14277980

[B28] Cantrelle P Mortalité infanto-juvénile d’hivernage dans le Sine-Saloum Environ Afr 1980 4 14–16 413 428 12146286

[B29] Cantrelle P Leridon H Breastfeeding, mortality in childhood and fertility in rural zone of Senegal Popul Stud 1971 25 3 505 533 10.1080/00324728.1971.10405821 22070150

[B30] Garenne M Maire B Fontaine O Dieng K Briend A Risques de décès associés à différents états nutritionnels chez l’enfant d’âge préscolaire Sérieétudeset theses 1987 ORSTOM, Paris

[B31] Briend A Garenne M Maire B Fontaine O Dieng K Nutritional status, age and survival: the muscle mass hypothesis Eur J Clin Nutr 1989 43 10 715 726 2612460

[B32] Garenne M Cantrelle P Mortalité des enfants ayant participé à un programme de protection nutritionnelle (Diohine, Sénégal) Estimation de la mortalité du jeune enfant (0–5 ans) pour guider les actions de santé dans les pays en développement 1986 INSERM 515 532

[B33] Ofusu-Amaah S Neumann A The Danfa Project 1979 University of Ghana Medical School & UCLA School of Public Health

[B34] Darras C Van Lerberghe W Mercenier P Le Project Kasongo: une expérience d’organisation d’un système de soins de santé primaries Ann Soc Belg Med Trop 1981 6 1 53 7340706

[B35] Mayombana C de Savigny D Tayari S Mutakyawa B Lubomba G Hatz C Village health workers in Kilombero district: training, performance and supervision7th Annual Scientific Conference: November 8th - 11th 1988 1988 Tanzania, Arusha, Tanzania

[B36] Tollman SM Zwi AB Health system reform and the role of field sites based upon demographic and health surveillance Bull World Health Organ 2000 78 1 125 134 10686747 PMC2560602

[B37] Sauerborn R Nougtara A Hien M Diesfeld HJ Seasonal variations of household costs of illness in Burkina Faso Soc Sci Med 1996 43 3 281 290 10.1016/0277-9536(95)00374-6 8844931

[B38] Binka FN Nazzar A Phillips JF The Navrongo Community Health and Family Planning Project Stud Fam Plann 1995 26 3 121 139 10.2307/2137832 7570763

[B39] Muhwava W Nyirenda M Mutevedzi T Herbst K Hosegood V Operational and Methodological Procedures of the Africa Centre Demographic Information System Monograph Series 2008 Africa Centre for Health and Population Studies 10.1093/ije/dym211 PMC2557060 17998242

[B40] Sankoh O Byass P The INDEPTH Network: filling some international gaps in epidemiology Int J Epidemiol 2012 41 3 579 588 in press 10.1093/ije/dys081 22798690 PMC3396316

[B41] Gladwin J Dixon RA Wilson TD Implementing a new health management information system in Uganda Heath Policy Plan 2003 18 2 214 224 10.1093/heapol/czg026 12740326

[B42] Arudo J Gimnig JE TerKuile FO Kachur SP Slutsker L Kolczak MS Hawley WA Orago ASS Nahlen BL Phillips-Howard PA Comparison of government statistics and demographic surveillance to monitor mortality in children less than five years old in Rural Western Kenya AmJTrop Med Hyg 2003 68 Suppl 4 30 37 12749483

[B43] de Savigny D Setel P Kasale H Whiting D Reid G Kitange H Mbuya C Mgalula L Machibya H Kilima P Demographic Surveillance and Health Service Needs: The AMMP/TEHIP experience in Morogoro, Tanzania 1999 Presented at the Multilateral Initiative on Malaria Conference, Durban, South Africa http://research.ncl.ac.uk/ammp/site_files/public_html/mim.pdf

[B44] de Savigny D Kasale H Mbuya C Reid G Fixing Health Systems.Published by the IDRC 2004 ISBN 1-55250-155-8, Ottawa, Canada

[B45] Byass P Sankoh O Tollman SM Högberg U Wall S Lessons from History or Designing and Validating Epidemiological Surveillance in Uncounted Populations PLoS One 2011 6 8 e22897 10.1371/journal.pone.0022897 21826215 PMC3149617

[B46] Byass P Worku A Berhane EY DSS and DHS: longitudinal and cross-sectional viewpoints on child and adolescent mortality in Ethiopia Popul Health Metr 2007 5 12 10.1186/1478-7954-5-12 18162133 PMC2235826

[B47] Navrongo Health Research C Where did CHPS come from? What works? What fails? Findings from the Navrongo Community Health and Family Planning Project. Volume 2 2002 Navrongo Health Research Centre, Ghana 1 2

[B48] Russell S Community-based Health Planning and Services (CHPS): Decentralizing Ghana’s Health System GUJHS 2008 5 1 http://www.jhuccp.org/africa/com_mob/ghana_chps.shtml

[B49] Floyd S Molesworth A Dube A Banda E Jahn A Mwafulirwa C Ngwira B Branson K Crampin AC Zaba B Glynn JR French N Population-level reduction in adult mortality after extension of free anti-retroviral therapy provision into rural areas in northern Malawi PLoS One 2010 5 10 e13499 10.1371/journal.pone.0013499 20976068 PMC2957442

[B50] Jahn A Floyd S Crampin AC Mwaungulu F Mvula H Munthali F McGrath N Mwafilaso J Mwinuka V Mangongo B Fine PE Zaba B Glynn JR Population-level effect of HIV on adult mortality and early evidence of reversal after introduction of antiretroviral therapy in Malawi Lancet 2008 371 1603 1611 10.1016/S0140-6736(08)60693-5 18468544 PMC2387197

[B51] Gilks CF Crowley S Ekpini R Gove S Perriens J Souteyrand Y Sutherland D Vitoria M Guerma T De Cock K The WHO public-health approach to antiretroviral treatment against HIV in resource-limited settings Lancet 2006 368 505 510 10.1016/S0140-6736(06)69158-7 16890837

[B52] Baiden F Hodgson A Binka FN Demographic Surveillance Sites and emerging challenges in international health Bull World Health Organ 2006 84 3 163 10.2471/BLT.05.025577 16583067 PMC2627292

[B53] Dodowa DSS http://www.indepth-network.org/dss_site_profiles/DodowaProfile2006.pdf

[B54] Manhica Demographic Surveillance System, Mozambique Manhica Demographic Surveillance System, Mozambiquehttp://www.indepth-network.org/dss_site_profiles/manhicadss.pdf

[B55] CDC/KEMRI DSS, Kisumuhttp://www.indepth-network.org/dss_site_profiles/kisumuprofile.pdf

[B56] KEMRI CGMRC Kilifi, Kenya [http://www.indepth-network.org/dss_site_profiles/KILIFI%20PROFILE%202006.pdf]

[B57] Nouna DSS, Burkina Faso http://www.indepth-network.org/Profiles/Nouna%20HDSS.pdf

[B58] Bandim DSS, Guinea-Bissauhttp://www.indepth-network.org/dss_site_profiles/bandim.pdf

[B59] Rommelmann V Setel PW Hemed Y Angeles G Mponezya H Whiting D Boerma T Cost and Results of Information Systems for Health and Poverty Indicators in the United Republic of Tanzania Bull World Health Organ 2005 83 8 569 577 16184275 PMC2626321

